# Influence of local aortic calcification on periaortic adipose tissue radiomics texture features—a primary analysis on PCCT

**DOI:** 10.1007/s10554-022-02656-2

**Published:** 2022-06-25

**Authors:** Hishan Tharmaseelan, Matthias F. Froelich, Dominik Nörenberg, Daniel Overhoff, Lukas T. Rotkopf, Philipp Riffel, Stefan O. Schoenberg, Isabelle Ayx

**Affiliations:** 1 Department of Radiology and Nuclear Medicine, University Medical Center Mannheim, Heidelberg University, Theodor-Kutzer-Ufer 1-3, 68167 Mannheim, Germany; 2 Department of Diagnostic and Interventional Radiology and Neuroradiology, Bundeswehr Central Hospital Koblenz, Rübenacher Straße 170, 56072 Koblenz, Germany; 3 Department of Radiology, German Cancer Research Center, Im Neuenheimer Feld 280, 69120 Heidelberg, Germany

**Keywords:** Photon-counting computed tomography, Periaortic adipose tissue, Texture analysis, Radiomics

## Abstract

Perivascular adipose tissue is known to be metabolically active. Volume and density of periaortic adipose tissue are associated with aortic calcification as well as aortic diameter indicating a possible influence of periaortic adipose tissue on the development of aortic calcification. Due to better spatial resolution and signal-to-noise ratio, new CT technologies such as photon-counting computed tomography may allow the detection of texture alterations of periaortic adipose tissue depending on the existence of local aortic calcification possibly outlining a biomarker for the development of arteriosclerosis. In this retrospective, single-center, IRB-approved study, periaortic adipose tissue was segmented semiautomatically and radiomics features were extracted using pyradiomics. Statistical analysis was performed in R statistics calculating mean and standard deviation with Pearson correlation coefficient for feature correlation. For feature selection Random Forest classification was performed. A two-tailed unpaired t test was applied to the final feature set. Results were visualized as boxplots and heatmaps. A total of 30 patients (66.6% female, median age 57 years) were enrolled in this study. Patients were divided into two subgroups depending on the presence of local aortic calcification. By Random Forest feature selection a set of seven higher-order features could be defined to discriminate periaortic adipose tissue texture between these two groups. The t test showed a statistic significant discrimination for all features (p < 0.05). Texture changes of periaortic adipose tissue associated with the existence of local aortic calcification may lay the foundation for finding a biomarker for development of arteriosclerosis.

**Supplementary Information:**

The online version contains supplementary material available at 10.1007/s10554-022-02656-2.

## Introduction

Cardiovascular diseases remain the leading cause of mortality and morbidity in developed countries [1] whereas arteriosclerosis counts as a leading cause of vascular diseases [2] and a vital cardiovascular disease risk marker [3, 4]. During the last years, incidence and mortality declined through the change in health behaviors and treatment for specific risk factors (i.e. hypercholesterolemia and hypertonia). Vascular mortality rates decreased from 700 per 100.000 per year in 1950 to < 100 per 100.000 per year in 2010 in middle-aged men in the United Kingdom [2]. Nevertheless, increasing efforts are needed to guarantee a further decline in incidence and mortality.

Specific adipose tissue depots have an association with cardiovascular diseases including subclinical atherosclerosis [5–9]. Especially perivascular adipose tissue, defined as fat deposits immediately surrounding blood vessels, is known to be metabolically active [7, 10–14]. Several studies have demonstrated a relationship between the volume and density of epicardial adipose tissue and the burden of coronary artery sclerosis [15–17], suggesting a local inflammatory effect on the vessel wall [9, 18, 19]. Additionally, the volume of the periaortic adipose tissue surrounding the descending thoracic aorta is associated with abdominal aortic calcification and coronary artery calcification [20]. The volume of periaortic adipose tissue surrounding the abdominal aorta was even associated with the aortic diameter outlining a potential effect on aortic remodeling and the development of an aortic abdominal aneurysm [21].

In the last few years, the main focus lay on the volumetric analysis of periaortic adipose tissue. However, initial results concerning the peri-coronary adipose tissue showed significant differences in the texture parameters in patients with and without plaques in the right coronary artery [22]. Radiomics feature analysis is an emerging technique that can extract pixel-based information from images to create datasets of hundreds of parameters [23–25]. Those features can be subdivided into the group of first-order statistics, which only describe the distribution of voxel without considering the spatial relationship, and the group of higher-order statistics summarizing texture features, shape-based parameters, and transform-based parameters. Texture features define the spatial distribution of voxels and hence visualize the heterogeneity of the area of interest [26]. This allows the extraction of image characteristics not visible to the human eye [25, 27] using dedicated software packages [28]. In recent years, Radiomics played a crucial role in the field of oncologic imaging and tumor analysis in terms of tumor classification [29–31] and outcome prediction [32, 33]. First analyses even support the strength of texture analysis in perivascular fat for risk stratification of cardiovascular patients [34]. However, a relevant limitation of radiomics evaluation in clinical routine is the need for a sufficient signal-to-noise ratio and optimal spatial resolution [35–37]. Through the implementation of photon-counting computed tomography (PCCT) these limitations could be overcome. The PCCT converts in contrast to conventional CT X-ray photons directly into an electric pulse without the intermediate step of converting them to visible light, so each photon contributes to the final visible image. This results in a better spatial resolution, a more sufficient contrast to noise ratio, and lower beam-hardening artifacts [38, 39].

The aim of the study is hence to analyze whether texture changes of periaortic adipose tissue depending on calcification of abdominal aorta can be revealed by texture analysis using PCCT, outlining a potential early biomarker for the development of arteriosclerosis.

## Materials and methods

### Study design

For this retrospective single-center study patients with clinically indicated thoracic and abdominal CT were enrolled between December 2021 and March 2022. In total 30 patients (10 males, 20 females, mean age 57 years, range: 23–84 years) were selected. All patients were examined using a clinically approved first-generation whole-body dual-source Photon-counting CT system. Patients were excluded in case of insufficient image quality (n = 3) or in case of metal artifacts (n = 2). Additionally, abdominal scans were screened for computer tomographic signs of mesenteric panniculitis or chronic pancreatitis by a radiologist with 9 years of experience (I.A.) and excluded in case of visible periaortic adipose tissue alterations. The electronic health record was reviewed for all patients to exclude diseases possibly affecting retroperitoneal adipose tissue or the vessels themselves. All investigations were conducted according to the Declaration of Helsinki. The retrospective study had an institutional review board approval. Table 1 shows an overview of the patient’s characteristics, as well as scan characteristics.

**Table 1:** Patient collective overview.

	Overall	No calcification	Calcification	p value
Patient parameters				
n	30	15	15	N/A
Age	57 (12.8)	50 (11.09)	63 (10.96)	0.002
Sex	10 male (33.3%)	4 male (26.7%)	6 male (40.0%)	0.699
Aortic abdominal calcifications	15/30	0/15	15/15	N/A
Calcification volume in mm^3^		0	1181.74 (1333.33)	N/A
Nicotine abuse	4/29	1/14	3/15	0.642
Hypertonia	8/29	0/14	8/15	0.005
Hyperlipidemia	5/29	0/14	5/15	0.060
Diabetes mellitus	5/29	0/14	5/15	0.060
Scanner parameters				
Tube voltage	120 kV	120 kV	120 kV	N/A
Slice thickness	1.5 mm	1.5 mm	1.5 mm	N/A
Kernel	Br40	Br40	Br40	N/A
Collimation	0.4 mm	0.4 mm	0.4 mm	N/A
Dose modulation	CARE Dose4D	CARE Dose4D	CARE Dose4D	N/A
Detector	PCD	PCD	PCD	N/A

Mean and (SD) given for continuous variables

### Abdominal CT acquisition protocol

All 30 patients were examined using the first whole-body dual-source Photon-counting computer tomography (NAEOTOM Alpha; Siemens Healthcare GmbH, Forchheim, Germany). The contrast-enhanced scan was performed using weight-adapted 70–90 ml of iodine contrast agent (Imeron 400, Bracco Imaging Deutschland GmbH, Konstanz, Germany) followed by a 20 ml saline chaser (NaCl 0.9%) with a weight-based flow rate via antecubital venous access. Bolus tracking was used to trigger the start of the arterial contrast phase of the thoracic organs by placing a region of interest (ROI) in the descending thoracic aorta (threshold 140 HU at 90 kV). An additional scan of the abdominal organs was done 75 s post-threshold.

### Abdominal CT reconstruction

The axial slices with 1.5 mm slice thickness and 1.5 mm spacing of the contrast-enhanced portal venous abdominal CT scan were exported from PACS and anonymized. Window-level and -width were determined using the standard window-level setting from the clinical routine.

### Image evaluation parameters and segmentation

**Fig. 1:**
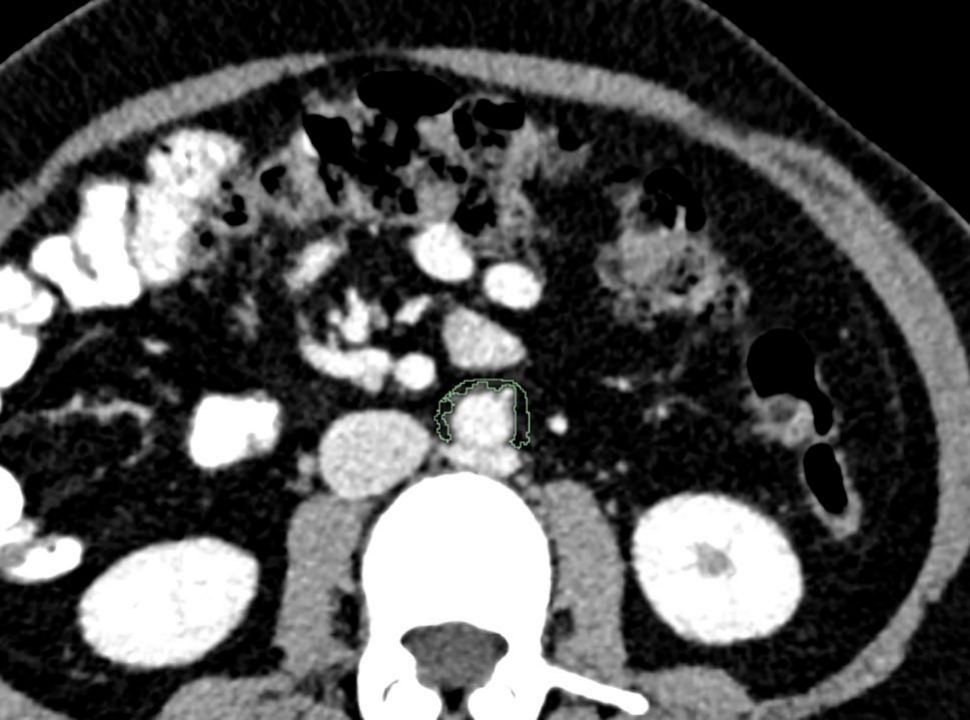
Segmentation of the peri-aortic abdominal adipose tissue was performed on axial view with a slice thickness of 1.5 mm. An example case of a 65-year old female patient is shown

For each patient, abdominal aortic calcification was diagnosed based on computed tomography by a clinically experienced radiologist on specially reconstructed virtual non-contrast enhanced images (I.A.), resulting in a classification of patients into two groups with and without abdominal aortic calcification. Segmentations were performed in the open-source software 3DSlicer by a medical student (H.T. with 2 years of experience in segmentation) (Version 4.11) [40]. Abdominal periaortic adipose tissue (AAT) was defined as any voxel between −195 and −45 HU as it was done in previous studies [41]. For segmentation a ring of 5 mm was drawn around the abdominal aorta including the defined voxels (Fig. 1). The region of measurement ranged from below the junction of the renal arteries with the abdominal aorta to the aortoiliac bifurcation. For volumetric evaluation of calcification, the calcifications of the infrarenal aorta were segmented.

### Radiomics feature extraction and statistical analysis

Radiomics features were extracted using pyradiomics, an imaging biomarker standardization initiative definition-based Python package [28]. First-order features and second-order features gray level co-occurence matrix (glcm), gray level size zone matrix (glszm), gray level run length matrix (glrlm), neighbouring gray tone difference matrix (ngtdm), and gray level dependence matrix (gldm) as defined in the pyradiomics documentation were extracted.

The statistical analysis of the calculated features was performed in R [42] and RStudio (version 1.3.1093, Boston, MA, USA) [43]. Mean and standard deviation values of quantitative parameters were calculated.

Radiomics feature extraction produces a vast amount of features that show high levels of redundancy. The random forest (RF) algorithm-based R package Boruta [44] was used to identify the most important features and plot the permutation-based variable importance.

Using the python package Complex Heatmap [45] all patients and features were plotted in a hierarchically clustered heatmap by euclidean distance. The most important features determined by the Boruta RF-feature selection were analyzed for significant differences using an unpaired two-tailed t-test. Additionally, the selected final features were visualized in boxplot diagrams.

## Results

### Cluster analysis

**Fig. 2:**
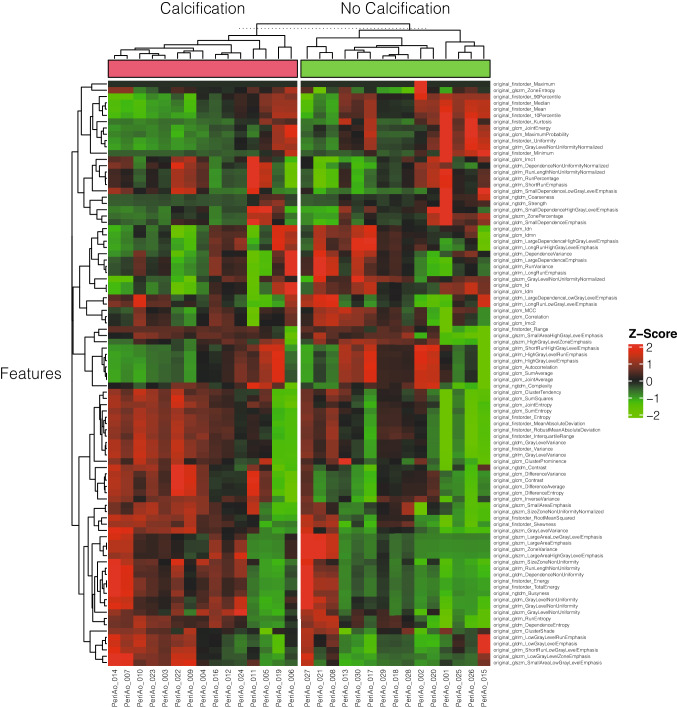
Unsupervised clustered heatmap for patients with or without abdominal aortic calcification

Unsupervised hierarchical clustering based on the euclidean distance between radiomics features extracted from the periaortic adipose tissue of each patient was performed after standardization. The heatmap was split into radiomics signatures of 15 patients with and 15 patients without calcification of the abdominal aorta. These results were visualized in a heatmap (Fig. 2).

### Feature selection

**Fig. 3:**
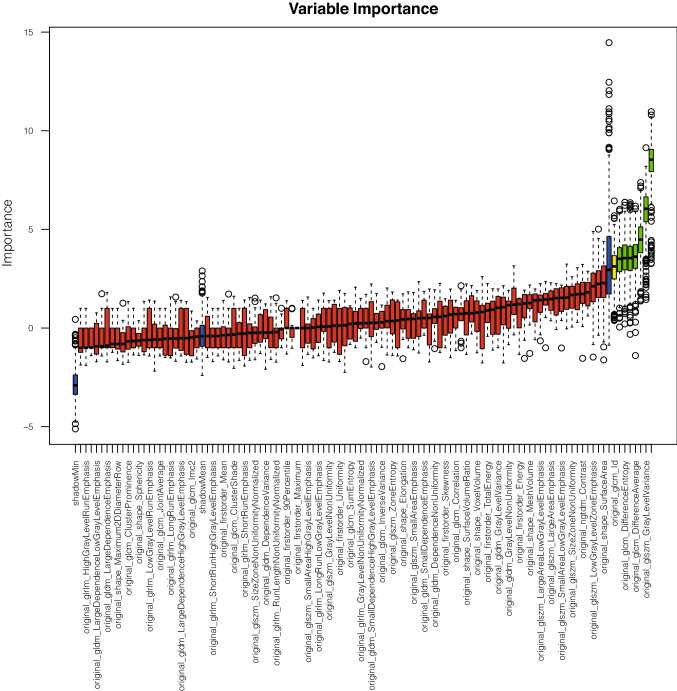
Random Forest permutation-based feature importance

Important features for the differentiation of patients were selected based on peri-aortic abdominal adipose tissue texture using Boruta/Random Forest-based feature selection. The selected features from patients with calcification and without calcification in the abdominal aorta showed seven second-order features as the most important for differentiation between both groups: “glcm_Contrast”, “glcm_DifferenceVariance”, “glcm_DifferenceAverage”, “glcm_DifferenceEntropy”, “glcm_JointEntropy”, “glszm_GrayLevelVariance”, and “glcm_MaximumProbability” (Fig. 3). GLCM and GLSZM features describe the distribution of intensity values. The exact ranking can be found in supplemental material S2. Furthermore, distortion of features by beam-hardening artifacts of aortic calcifications can be excluded in radiomics texture maps.

### Statistical analysis

**Fig. 4:**
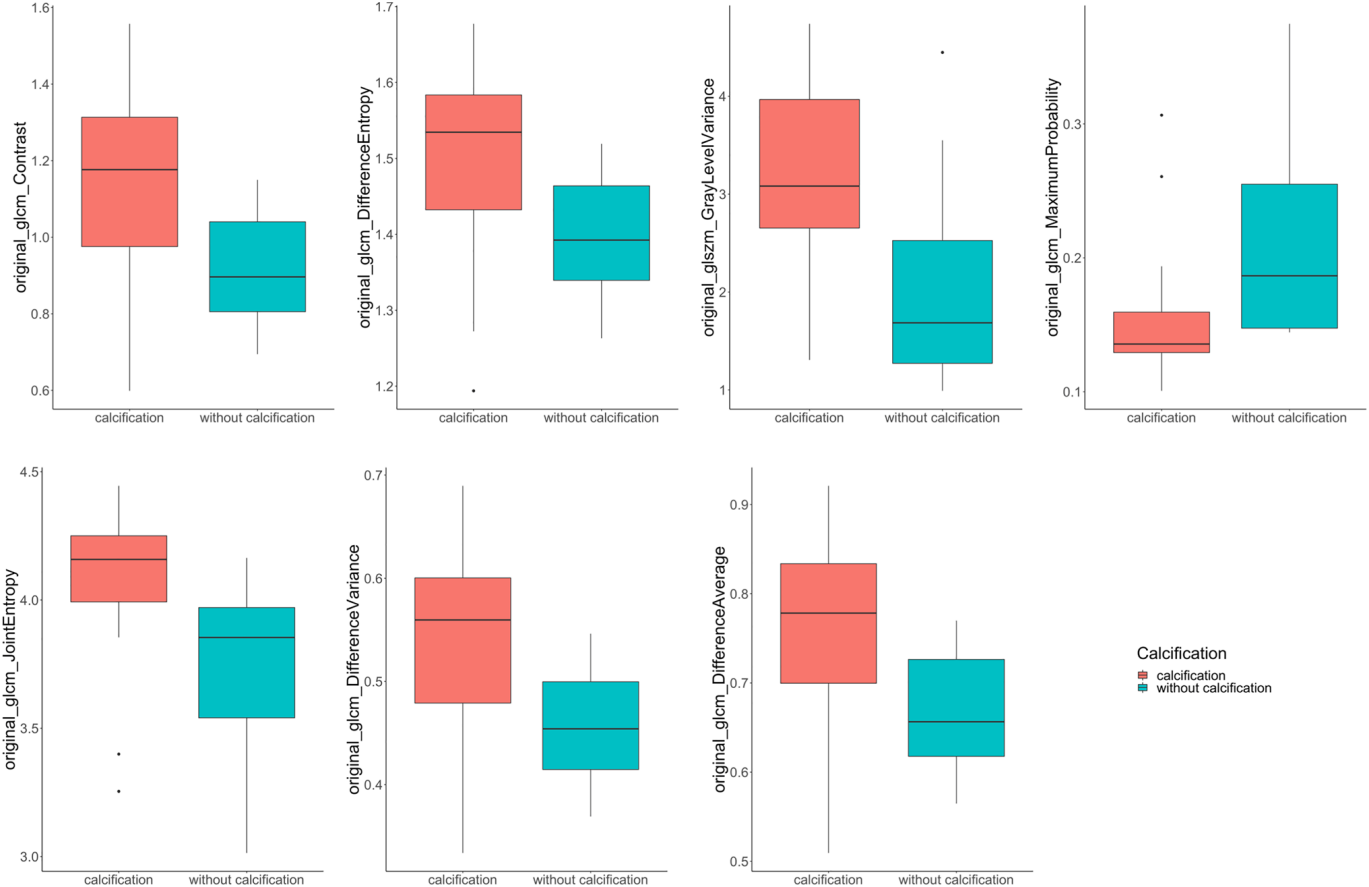
Distribution of “glcm_Contrast”, “glcm_DifferenceVariance”, “glcm_DifferenceAverage”, “glcm_DifferenceEntropy”, “glcm_JointEntropy”, “glszm_GrayLevelVariance”, and “glcm_MaximumProbability”,”glcm_DifferenceEntropy” features within the dataset visualized by a boxplot diagram

The final selection of features was tested for statistical significance between both groups using the two tailed—unpaired t-test. All of these features showed a significant difference (*p* < 0.05) in mean values: “glcm_Contrast” (*p* = 0.007), “glcm_DifferenceVariance” (*p* = 0.009), “glcm_DifferenceAverage” (*p* = 0.010), “glcm_DifferenceEntropy” (*p* = 0.015), “glcm_JointEntropy” (*p* = 0.014), “glszm_GrayLevelVariance” (*p* = 0.006), and “glcm_MaximumProbability” (p = 0.031) (Table 2). The boxplot diagram allows visualization of the significantly different features (Fig. 4). Volumetric analysis of calcification showed a value of 1181.74 (1333.33) mm^3^ (mean [SD]).

**Table 2:** Higher order radiomics features.

	Calcification	No calcification	p value
original_glcm_MaximumProbability	0.16 (0.06)	0.21 (0.07)	0.031
original_glszm_GrayLevelVariance	3.17 (1.10)	2.02 (1.00)	0.006
original_glcm_JointEntropy	4.05 (0.33)	3.74 (0.32)	0.014
original_glcm_DifferenceAverage	0.76 (0.11)	0.67 (0.07)	0.010
original_glcm_DifferenceEntropy	1.50 (0.13)	1.40 (0.08)	0.015
original_glcm_DifferenceVariance	0.54 (0.10)	0.46 (0.05)	0.009
original_glcm_Contrast	1.14 (0.27)	0.92 (0.15)	0.007

Mean and (SD) given for continuous variables

## Discussion

Our study demonstrates the association of texture features of periaortic adipose tissue with the presence of local abdominal aortic calcifications. Differentiation between patients with and without abdominal aortic calcifications was possible through seven different texture-related higher-order features, revealing a prediction of the presence of calcifications using radiomics analysis of periaortic adipose tissue solely, which may suggest that periaortic adipose tissue can be used as a biomarker for atherosclerosis.

The importance of perivascular adipose tissue has already been shown in recent studies but needs to be investigated further, particularly on the imaging basis. Several studies have demonstrated a relationship between the volume and density of epicardial adipose tissue and the burden of coronary artery sclerosis [15–17], suggesting a local inflammatory effect on the vessel wall [9, 18, 19].

The preliminary results of our study are in line with previously published studies: Lehmann et al. were able to show that the volume of periaortic adipose tissue surrounding the descending thoracic aorta is associated with abdominal aortic and coronary artery calcification [20]. Furthermore, it was shown that the volume of periaortic adipose tissue surrounding the abdominal aorta was associated with the aortic diameter outlining a potential effect on aortic remodeling and the development of an aortic abdominal aneurysm [21]. Shields et al. were able to demonstrate an association between a higher density of periaortic adipose tissue and arteriosclerosis in women suffering from systemic lupus erythematosus, possibly revealing an adipose dysfunction as well as visualization of fibrotic changes. Additionally, they revealed a correlation between higher density and volume of periaortic adipose tissue [46]. In line with these results, Alvey et al. found more dense visceral adipose tissue in association with coronary artery calcifications as well as arteriosclerosis, suggesting an excess collagen production in response to chronic inflammatory reaction [47]. In contrast to this, different studies revealed an association of lower dense adipose tissue with cardiovascular disease and cardiometabolic risk [48, 49]. Until now, there is still disagreement in the literature on whether higher or lower dense adipose tissue shows an influence on aortic calcification. Radiomics texture analysis can potentially overcome this disadvantage by generating a deeper insight. Through texture analysis special texture features of periaortic adipose tissue could be determined depending on the presence of focal aortic calcification, focusing not only on the measured density alone. This may allow a potential texture feature biomarker to be defined, which can possibly predict the development of aortic calcification in the future.

Through the implementation of PCCT the previous limitations of radiomics texture analysis in cardiac CT can be overcome by achieving better spatial resolution and contrast-to-noise ratio. The analysis of finer structures with PCCT may lay the foundation for determining such a potential imaging biomarker in the future, offering new possibilities of cardiovascular prevention. Additional analysis of the time course of structural changes in periaortic adipose tissue texture could help to identify underlying mechanisms and their power as a predictor. Yet, this work is the first to investigate texture features of periaortic adipose tissue in association with aortic calcifications in a photon-counting CT dataset. This may boost the development of early diagnostic tools for aortic arteriosclerosis and the implementation of periaortic adipose tissue texture as a biomarker for cardiovascular events. Especially in the context of machine learning modelings, first results in cardiovascular diseases have shown very promising results and could, besides the classical diagnostic applications, lead to a more precise cardiovascular risk stratification [50].

Finally, a number of potential limitations need to be considered in the interpretation of this work. This study was designed as a retrospective analysis of a small number of patients, due to the novel implementation of PCCT. Although this approach allows us to take advantage of the more accurate spatial resolution power of photon-counting CT which is new to clinical practice, further analysis would be necessary on a larger cohort to develop possible predictive models from this correlation. Limitations concerning the radiomics methodology, especially reproducibility [24], need to be considered but have been addressed by a single-scanner approach using a high-resolution detector. Further studies should also regard the limitation of not taking clinical characteristics as well as atheromatosis into account. Additionally, further prospective studies with a larger population should outline the benefit of periaortic adipose tissue as a biomarker for arteriosclerosis especially over time on a longitudinal study.

In conclusion, this study outlines the effect of texture changes of the periaortic adipose tissue on development of local aortic calcifications generating the hypothesis of a possible structural change through inflammatory and fibrotic processes. The preliminary results of this study may pave the way for additional studies revealing an imaging biomarker for early prediction of arteriosclerosis.

## Supplementary Information

Below is the link to the electronic supplementary material.

Supplementary file1 (DOCX 17 kb)

## Abbreviations

AAT: Abdominal periaortic adipose tissue
CT: Computed tomography
GLCM: Grey level co-occurence matrix
GLDM: Grey level dependence matrix
GLRLM: Grey level run length matrix
GLSZM: Grey level size zone matrix
NGTDM: Neighboring grey tone difference matrix
PCCT: Photon-counting computed tomography
RF: Random forest
ROI: Region of interest
