# Supplementary material for: Influence of local aortic calcification on periaortic adipose tissue radiomics texture features—a primary analysis on PCCT
Source: Int J Cardiovasc Imaging. 2022 Jun 25;38(11):2459–67. doi: 10.1007/s10554-022-02656-2 (PMC9700618; doi:10.1007/s10554-022-02656-2)
Supplement: Supplementary file 1 — Supplementary file1 (DOCX 17 kb) [file 10554_2022_2656_MOESM1_ESM.docx]

**Supplementary**

**S1: Pyradiomics Settings**

*Extraction parameters:*

{'minimumROIDimensions': 2, 'minimumROISize': None, 'normalize': False, 'normalizeScale': 1, 'removeOutliers': None, 'resampledPixelSpacing': None, 'interpolator': 'sitkBSpline', 'preCrop': False, 'padDistance': 5, 'distances': [1], 'force2D': False, 'force2Ddimension': 0, 'resegmentRange': None, 'label': 1, 'additionalInfo': True}

Enabled filters: {'Original': {}}

Enabled features: {'firstorder': [], 'glcm': [], 'gldm': [], 'glrlm': [], 'glszm': [], 'ngtdm': [], 'shape': []}

**S2: Boruta mean importance output**

|  | meanImp | decision |
| --- | --- | --- |
| original_glcm_MaximumProbability | 8.440041 | Confirmed |
| original_glszm_GrayLevelVariance | 5.5961439 | Confirmed |
| original_glcm_JointEntropy | 4.450552 | Confirmed |
| original_glcm_DifferenceAverage | 3.589867 | Confirmed |
| original_glcm_DifferenceEntropy | 3.558043 | Confirmed |
| original_glcm_DifferenceVariance | 3.519863 | Confirmed |
| original_glcm_Contrast | 3.450324 | Confirmed |
